# Quantitative Autofluorescence Imaging of Oral Mucosa and Lesions: A Proof-of-Concept Study

**DOI:** 10.3390/diagnostics16060857

**Published:** 2026-03-13

**Authors:** Keerthi Gurushanth, Sumsum P. Sunny, Shubha Gurudath, Harshita Thakur, Kripa Adlene Edith, Keerthi Krishnakumar, Shikha Jha, Pavitra Chandrashekhar, Satyajit Topajiche, Lynette Linzbuoy, Sanjana Patrick, Ramyashree Rao, Simranjeet Kaur, Umeshgouda Patil, Ananya Nagaraj, Bofan Song, Rongguang Liang, Shubhasini Raghavan, Anupama Shetty, Amritha Suresh, Moni Abraham Kuriakose, Praveen Birur Nagaraj

**Affiliations:** 1 Department of Oral Medicine and Radiology, KLE Society’s Institute of Dental Sciences, Bengaluru 560022, India; keerthi_g80@yahoo.com (K.G.); shubha.gurudath@gmail.com (S.G.); ht49701@gmail.com (H.T.); edithkripa3@gmail.com (K.A.E.); keerthikkumar95@gmail.com (K.K.); shikhajha298@gmail.com (S.J.); ramyashreerao22@gmail.com (R.R.); ksimranjeet151@gmail.com (S.K.); umeshspatil1997@gmail.com (U.P.); ananyanagaraj98@gmail.com (A.N.); subhashiniar@gmail.com (S.R.); 2 Integrated Head and Neck Oncology Program, Mazumdar Shaw Medical Foundation, Narayana Health City, Bommsandra Industrial Area, Bengaluru 560099, India; sumsumsp@gmail.com (S.P.S.); amritha.suresh@ms-mf.org (A.S.); 3 Department of Oral Pathology, KLE Society’s Institute of Dental Sciences, Bengaluru 560022, India; drpavitra.c@gmail.com (P.C.); drtopajiche@gmail.com (S.T.); 4 Biocon Foundation, Bengaluru 560099, India; lynette.linzbuoy@gmail.com (L.L.); tanjupat@yahoo.com (S.P.); anupama.shetty101@biocon.com (A.S.); 5 College of Optical Sciences, The University of Arizona, Tucson, AZ 85721, USA; songbofan@gmail.com (B.S.); rliang@optics.arizona.edu (R.L.); 6 Karkinos Healthcare, Kochi 682017, India; makuriakose@gmail.com

**Keywords:** autofluorescence imaging, intensity, oral precancer, oral cancer, oral mucosa, quantitative

## Abstract

**Background/Objectives:** This study aimed to quantitatively assess site-specific mean autofluorescence intensity across normal oral mucosal subsites and to evaluate the effectiveness of Autofluorescence Imaging (AFI) as an adjunct tool for distinguishing benign lesions, OPMDs, and oral cancers by comparing lesion intensity with anatomically matched healthy subsites. **Methods:** This observational study employed dual-mode imaging, comprising paired White Light Imaging (WLI) and AFI, captured from different oral cavity subsites using a smartphone-based point-of-care device. The Region of Interest (ROI) was annotated on WLI and automatically mapped to the corresponding AFI for both normal mucosa and lesions. WLI and AFI images were separated into their constituent red, green, and blue (RGB) channels, and AFI intensity was quantified via ImageJ. **Results:** A total of 1380 dual-mode images were acquired from 86 healthy participants. AFI intensities were comparable across most oral subsites, except for the lateral and ventral tongue. The lateral border showed the lowest fluorescence (Green channel-GC: 68.12 ± 28.27; Blue channel-BC: 25.29 ± 7.93), whereas the ventral tongue showed the highest (GC: 98.89 ± 42.22; BC: 37.08 ± 11.04; both *p* < 0.001). Among 611 lesions, predominantly from the buccal mucosa, AFI intensity declined progressively with increasing disease severity. Homogeneous leukoplakia (*n* = 149; GC: 38.62 ± 25.05; BC: 21.60 ± 9.50), non-homogeneous leukoplakia (*n* = 25; GC: 30.42 ± 18.66; BC: 18.25 ± 7.17) and oral cancer (*n* = 21; GC: 23.39 ± 15.53; BC: 15.82 ± 7.15; all *p* < 0.001) showed markedly reduced fluorescence, while benign lesions (*n*: 44; GC: 66.99 ± 30.88; BC: 32.01 ± 13.62) exhibited intermediate intensities, supporting AFI’s discriminative potential. **Conclusions:** This phase-1, proof-of-concept study highlights subsite-specific variations in autofluorescence intensity within healthy oral mucosa, providing an essential baseline for objective interpretation of lesion-associated fluorescence changes. AFI has the potential to be used as a non-invasive adjunct for monitoring OPMDs. Further validation in larger and more diverse cohorts is required before clinical implementation.

## 1. Introduction

Lip and oral cavity cancer is the 16th most common cancer worldwide, with Asia accounting for over two-thirds of new cases and related deaths [1,2]. Because more than 80% of oral cancers arise from clinically evident yet asymptomatic Oral Potentially Malignant Disorders (OPMDs), early detection is critical for improving outcomes [3,4,5,6]. Conventional diagnosis relies on oral visual tactile examination and biopsy confirmation, but population-level screening is limited by variable diagnostic accuracy, restricted access in low-resource settings, and the invasive nature of biopsies, which may reduce patient compliance [4,5,7,8]. To address these challenges, recent strategies have integrated AI-enabled mHealth platforms with optical diagnostic adjuncts to facilitate noninvasive, point-of-care screening [9,10]. Our team previously demonstrated the feasibility of a smartphone-based dual-mode device combining polarized White Light Imaging (WLI) and Autofluorescence Imaging (AFI) [11,12]. While WLI simulates routine clinical inspection, AFI enables detection of dysplasia through fluorescence visualization loss (FVL), in contrast to fluorescence visualization retention (FVR) in healthy mucosa [4,11,13].

A recent systematic review and meta-analysis evidence reports a moderate pooled sensitivity (0.75; 95% CI: 0.72–0.78) and low specificity (0.50; 95% CI: 0.47–0.53) for AFI in differentiating dysplastic from nondysplastic regions [10]. This lower specificity is largely attributed to confounding factors, such as inflammatory, vascular, pigmented, and benign lesions, which can mimic dysplasia under AFI [13]. We hypothesize that an additional and underexplored factor is the absence of fluorescence reference values. Autofluorescence varies across oral subsites due to differences in epithelial thickness, keratinization, vascularity, and surface morphology [14]; therefore, establishing baseline fluorescence intensities is essential for accurately interpreting lesion-associated changes.

To the best of our knowledge, no systematic and quantitative assessment of subsite-specific autofluorescence intensities in healthy oral mucosa has been reported while accounting for anatomical heterogeneity. Thus, the primary objective of this phase-1, proof-of-concept study was to establish baseline autofluorescence values across normal oral subsites to inform future large-scale investigations. The secondary objective was to evaluate the ability of AFI to discriminate between benign lesions, OPMDs, and oral cancers by comparing lesion-associated intensities with anatomically matched healthy subsites using a smartphone-based dual-mode imaging tool.

## 2. Materials and Methods

### 2.1. Imaging Device and Standardization

**Figure 1:**
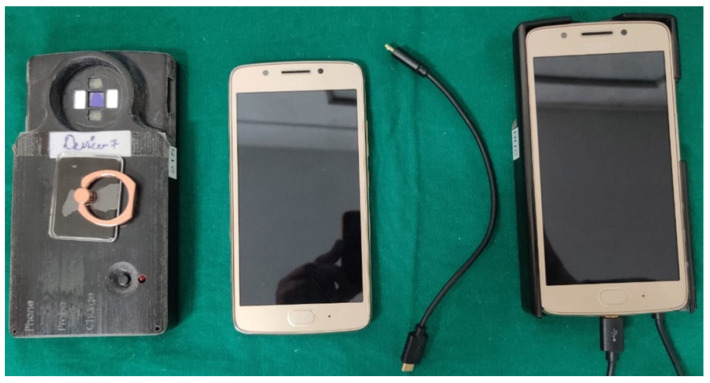
Point-of-care device.

A smartphone-based dual-mode point-of-care imaging tool was used to capture WLI and AFI images from all oral cavity subsites (Figure 1). The system incorporated a commercially available Moto G5 android smartphone (Motorola Inc., Schaumburg, IL, USA; Android version 7), enabling portable imaging, onboard computation, data transmission, and a touchscreen interface for user-friendly operation. The device integrated both Autofluorescence Imaging (AFI; excitation 400–410 nm) and polarized White Light Imaging (WLI), allowing dual-mode acquisition with a single click for each subsite.

The small numerical aperture of the smartphone camera provided a long depth of field, enabling simultaneous visualization of a wide anatomical region. The camera (13 MP; 1920 × 1080 display resolution) operated with a fixed focus and was positioned at a standardized distance from the oral cavity entrance, as deviations resulted in image blurring. A distance of 6–8 cm was used for anterior oral subsites and 8–10 cm for posterior subsites. These ranges were established after trials to take intraoral photographs and were found to provide optimal image quality while minimizing blur; distances outside these ranges consistently produced loss of sharpness. Specifying these values ensures reproducibility of the imaging protocol. Additionally, an auto-feedback mechanism continuously monitored sharpness and focus metrics, alerting the operator if the device was positioned too close or too far. After each capture, an automated “good” or “poor” image-quality notification further ensured consistent and reproducible image acquisition.

The device adhered to standardized calibration procedures established and validated in our previous work [11,12]. Quantitative calibration included (i) illumination uniformity, measured on a matte white target as 1 − coefficient of variation after relative-illumination correction, with a target ≥0.90 and achieved values of 0.96 for both white-light and AF channels (~4% variation across the field of view), and (ii) spectral output, measured at the tissue plane using a calibrated fiber-coupled spectrometer [11]. The AF channel produced excitation centered at ~405 nm with detection through a 470 nm long-pass filter, while the white-light channel showed a 450–650 nm emission spectrum. These measured spectra guided LED drive current and exposure settings and were consistent with manufacturer specifications [11,12]. All operators underwent structured training in subsite identification, device positioning, handling, and maintenance of consistent imaging conditions, thereby enhancing the reproducibility of the acquisition protocol.

### 2.2. Study Design and Image Analysis

A two-step methodological approach was implemented:

#### 2.2.1. Objective Assessment of Autofluorescence Intensity for Different Subsites of Normal Oral Mucosa

This phase-1 study aimed to establish baseline, subsite-specific autofluorescence intensity values in healthy oral mucosa to support future large-scale and AI-assisted investigations. Specifically, the study sought to objectively quantify the fluorescence intensity of AFI images. The study adhered to the Declaration of Helsinki, was approved by the Institutional Review Board of KLE Society’s Institute of Dental Sciences (KIDS/IEC/02-2021/05; 18 February 2021), and was registered with the Clinical Trial Registry of India (CTRI/2021/04/032536; 5 April 2021).

**Figure 2:**
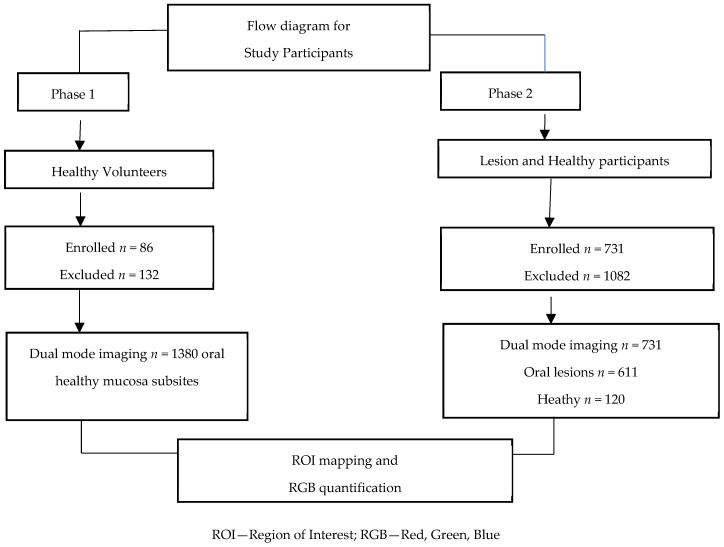
Flow diagram illustrates the participants enrollment for the study.

The flow diagram (Figure 2) illustrates the participants’ enrollment for the study. Healthy adults aged ≥18 years with no history of tobacco use (smoking or smokeless), arecanut consumption, alcohol intake, or clinical evidence of oral lesions were enrolled after obtaining written informed consent. Trained healthcare workers captured WLI and AFI images from all oral subsites, including lips, buccal mucosa, floor of the mouth, dorsal, ventral, and lateral tongue, gingiva, retromolar regions, hard and soft palate, and vestibules, in a systematic clockwise sequence.

**Figure 3:**
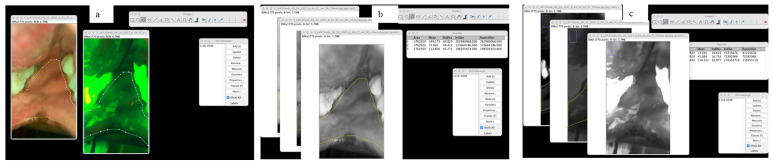
(**a**) shows an annotated white light image and corresponding autofluorescence image via ImageJ software; (**b**,**c**) shows the split red, green and blue channels for white light imaging and autofluorescence imaging, respectively.

Images were processed using ImageJ (version 2.3.0). WLI and AFI images were imported into the software by navigating the selected files to open the menu bar (Figure 3a), and anatomical boundaries of each subsite were manually annotated on WLI. The corresponding ROI was automatically mapped to AFI. Both the WLI and AFI images were subsequently split into their constituent Red, Green, and Blue (RGB) channels (Figure 3b,c). For both WLI and AFI, the area, mean, standard deviation, and intensity metrics, including the integrated density and raw integrated density values, were computed.

Channel-wise analysis enabled extraction of granular and physiologically relevant information, as each RGB channel reflects distinct dominant fluorophores and optical tissue characteristics. Mean pixel intensity captured average fluorescence independent of ROI size, whereas integrated density represented total fluorescence output, accounting for both ROI area and intensity.

##### Repeatability Assessment

To evaluate temporal consistency and device reproducibility, RGB intensity measurements were obtained at two timepoints one week apart under identical imaging conditions. Images were captured at a standardized perpendicular orientation using ambient dental-chair lighting, reflecting real-world screening conditions encountered by end users. A graduated probe was included in all images as a dimensional reference for lesion tracking during follow-ups. The reproducibility of the intensity measurements across two time points was assessed to determine the consistency of the imaging tool.

#### 2.2.2. Comparison of Lesion Intensity with Matched Healthy Subsites

The second component assessed the effectiveness of AFI for distinguishing benign lesions, OPMDs, and oral cancers by comparing lesion intensities with anatomically matched healthy subsites. Clinical classification of lesions was performed by an oral medicine specialist; however, histopathological confirmation was not available for all oral lesions. For this phase of the study, only the provisional clinical diagnosis was regarded as the reference standard. For each case, WLI and AFI images were imported into ImageJ. The lesion ROI was manually delineated on the WLI image and automatically transferred to the corresponding AFI using pixel-level registration. Manual annotation was initially performed by a single examiner; however, to further minimize inter-operator variability, two independent observers subsequently annotated all lesion ROIs, and any discrepancies were resolved through consensus on lesion margins and diagnosis. Although formal inter-rater reliability metrics were not calculated, the use of dual observers with consensus review helped reduce subjectivity in ROI delineation. Nevertheless, inter- and intra-observer reproducibility was not formally evaluated and is acknowledged as a limitation of the study. As previously described, WLI and AFI were split into RGB channels, and area, mean intensity, standard deviation, and integrated density were extracted. This approach enabled direct, subsite-specific comparison of lesion-related fluorescence with baseline healthy values, thereby supporting objective differentiation of pathological changes. The analysis targeted commonly affected sites: buccal mucosa, labial mucosa, palate, and tongue.

### 2.3. Statistical Analysis

Sample Size Estimation

A pilot analysis of 30 clinically normal oral sites evaluated differences in green-channel autofluorescence intensity. The observed mean difference between buccal mucosa and tongue was 13 (SD = 30). Using these parameters, a minimum sample size of 85 per subsite was calculated to achieve 80% power at *p* < 0.05. Ultimately, 86 participants were recruited to ensure adequate representation across major oral subsites, including buccal mucosa, tongue, labial mucosa, floor of the mouth, and gingiva.

Analytical Methods

Descriptive statistics summarized demographic and clinical characteristics. The normality of continuous variables was assessed using the Kolmogorov–Smirnov test. For subsite-level comparisons among healthy participants, independent or paired samples *t*-tests were applied to normally distributed data, and the Mann–Whitney U test was used for non-normally distributed data. As this was an exploratory analysis, multiple-comparison corrections were not applied; therefore, unadjusted *p*-values (*p* < 0.05) were reported.

Lesion-category comparisons (normal vs. benign vs. OPMD vs. cancer) were conducted using the Kruskal–Wallis test. Two-group comparisons were performed using either the independent/paired t-test (for normally distributed variables) or the Mann–Whitney U test (for non-parametric variables). A significance threshold of *p* < 0.05 was used.

Dimensionality reduction was performed with Principal Component Analysis (PCA) and t-Distributed Stochastic Neighbor Embedding (t-SNE), which served as exploratory visualization tools to assess clustering patterns and the distribution of fluorescence features. All statistical analyses were conducted using MedCalc (version 14.8.1). Data processing and visualization were performed using Python 3.7.4. including Sklearn, Computer Vision, Skimage, Matplotlib, NumPy, Pandas.

## 3. Results

### 3.1. Autofluorescence Intensity Across Oral Cavity Subsites in Healthy Individuals

**Figure 4:**
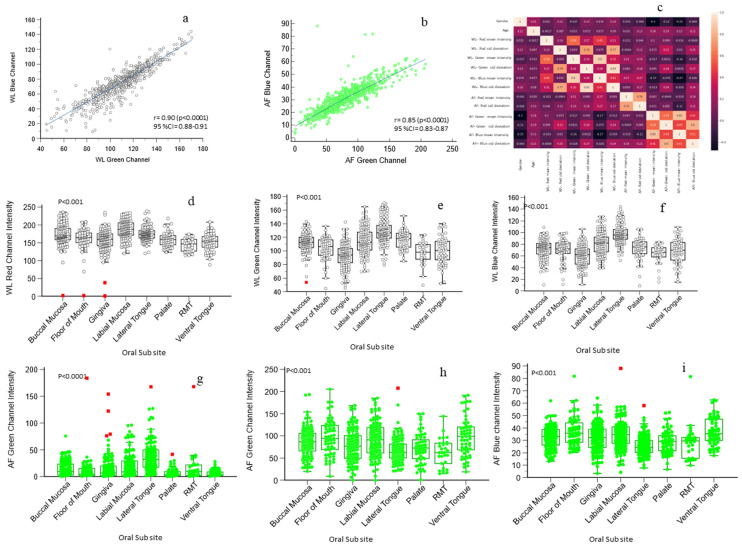
Distribution and correlation of RGB channel intensity (arbitrary units, 0–255). The green- and blue-channel intensities of white-light (WL) and autofluorescence (AF) images show strong correlations, ((**a**,**b**); r = Pearson correlation). The heat map of pairwise correlations between age, gender, and the mean and standard deviation of WL and AF RGB channel intensities (**c**). Box-and-whisker plots (**d**–**f**) depict the distribution of WL red, green and blue channel intensities across oral subsites, while (**g**–**i**) show the corresponding AF channel intensities, demonstrating significant inter-site differences. RMT = retromolar trigone; std = standard deviation; AF values are plotted as green dots and WL values as small white circle. The red dots represents the outliers.

A total of 1380 dual-mode images—comprising 690 WLI and 690 AFI images—were acquired from (*n* = 86) healthy participants (36 males, 50 females) aged 19–65 years. Images were obtained from eight oral subsites: buccal mucosa (*n* = 240), floor of the mouth (*n* = 120), gingiva (*n* = 240), labial mucosa (*n* = 240), lateral tongue (*n* = 240), palate (*n* = 120), retromolar trigone (*n* = 60), and ventral tongue (*n* = 120). Significant inter-subsite variability was observed across the red, green, and blue channel intensities. A strong linear correlation was identified between the green and blue channels in dual-mode images; therefore, these channels were evaluated in subsequent analyses (Figure 4a–c). Site-wise variations in WLI mean RGB intensities are shown in Figure 4d–f. The labial mucosa and the lateral tongue exhibited statistically significant differences across all three channels compared with the remaining subsites (*p* < 0.05; Supplementary Table S1).

In the AFI images, the green intensity values were highest for the ventral tongue [Green channel (GC): 98.89 ± 42.22) and floor of the mouth (GC:96.71 ± 39.66) and lowest for the retromolar trigone (GC:62.36 ± 31.38) and lateral tongue (GC:68.12 ± 28.28). The lateral tongue differed significantly (*p* < 0.05) from all other subsites. The blue channel intensity followed a similar trend, with the ventral tongue (37.08 ± 11.04) and floor of the mouth (37.12 ± 11.90) having the highest mean intensities.

The lateral tongue had the lowest blue intensity (Blue channel (BC): 25.30 ± 7.93), which was significantly lower than that at all subsites except the palate. Overall, the lateral tongue consistently demonstrated the lowest green and blue intensities and the highest red intensity, distinguishing it from other mucosal sites (*p* < 0.05). In contrast, the ventral tongue presented significantly greater green and blue intensity values while maintaining the lowest red intensity. Subsite-specific AFI mean intensities are summarized in Supplementary Table S2 and visualized in Figure 4g–i.

**Figure 5:**
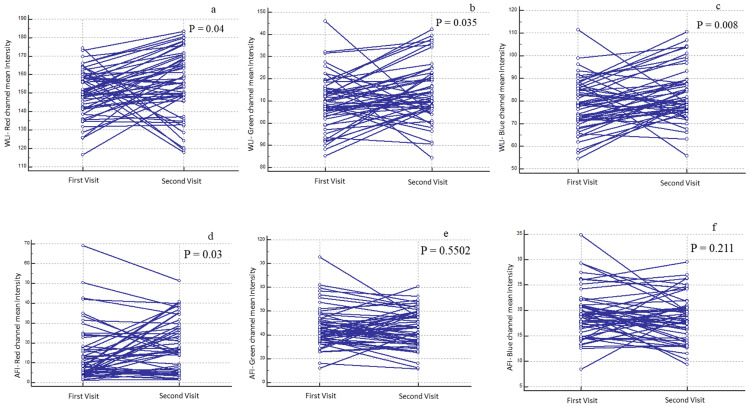
Paired comparison of RBG channel intensities of White Light Images (WLI) and Auto-Fluorescence Imaging (AFI). The paired dots plot shows the significant difference in first and second visit of WLI channels (**a**–**c**) and red channel (**d**) of AFI. The green and blue channel showed no difference between visits (**e**,**f**).

To evaluate temporal consistency, a total of (*n* = 480) images from healthy individuals were analyzed at two separate time intervals. The oral subsites imaged included the buccal mucosa (*n* = 120), floor of the mouth (*n* = 60), gingiva (*n* = 120), labial mucosa (*n* = 120), lateral tongue (*n* = 120), palate (*n* = 60), retromolar trigone (*n* = 30), and ventral tongue (*n* = 60). Paired *t*-tests revealed no significant temporal differences in mean RGB intensities for most subsites—including the buccal mucosa, tongue, and palate—indicating stable device performance (Supplementary Figures S1–S3). Significant changes were observed in WLI but not in AFI. For WLI, the GC channel increased from 107.57 ± 17.29 at the first visit (FV) (*n* = 57; (95% CI: 102.98–112.16) to 111.93 ± 18.29 (95% CI: 107.08–116.7) at the second visit (SV), corresponding to a 4.0% increase (*p* < 0.03). The BC channel increased from 76.84 ± 13.97 (FV) (95% CI: 73.13–80.55) to 81.60 ± 14.61 (SV) (95% CI: 77.73–85.48), indicating a 6.2% increase (*p* < 0.008) (Figure 5a–c). In contrast, AFI intensities showed no significant differences between visits (Figure 5d–f).

### 3.2. Evaluation of Effectiveness of AFI for Oral Lesion and Subsite-Specific RGB Intensity Variations

**Figure 6:**
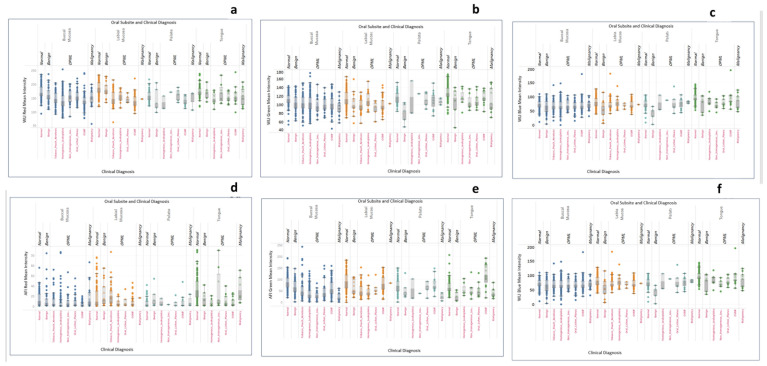
Distribution of RGB channel intensities in white-light images (WLI) and autofluorescence images (AFI) for site-wise and lesion-wise comparison. Colors indicate different oral subsites (buccal mucosa, labial mucosa, palate and tongue). The x-axis shows the clinical diagnosis, and the y-axis represents mean RGB pixel intensities for WLI (**a**–**c**) and AFI (**d**–**f**). OSMF = oral submucous fibrosis; OPML = oral potentially malignant lesions.

This study further assessed the ability of the AFI to distinguish between normal mucosa, benign lesions, OPMDs, and oral cancer, with a focus on subsite-specific variations in RGB intensity values. A total of (*n* = 731) images were analyzed, comprising normal tissue (*n* = 120) and lesion images (*n* = 611). The lesion cohort comprised benign lesions (*n* = 63), tobacco pouch keratosis (*n* = 114), oral submucous fibrosis (*n* = 146), oral lichen planus (*n* = 55), homogeneous leukoplakia (*n* = 171), non-homogeneous lesions (*n* = 32), and oral cancer (*n* = 30). Subsite-wise mean RGB intensities for WLI are presented in Figure 6a–c, and AFI intensities in Figure 6d–f.

Normal mucosa consistently displayed the highest mean intensities across RGB channels in both WLI and AFI (GC: 85.66; BC: 32.36), establishing an upper reference range. Lesions of all categories demonstrated reductions in one or more channels, particularly under AFI, indicating fluorescence visualization loss (FVL). Green and blue channel reductions under AFI are prominent and serve as useful indicators of lesion severity. The dataset predominantly comprised buccal mucosa lesions; hence, AFI intensity patterns were evaluated for this subsite. Most lesion subtypes showed a marked reduction in AFI green and blue intensities, reflecting loss of autofluorescence typically associated with epithelial dysplasia and inflammatory changes. AFI intensity declined progressively with increasing disease severity. Among 611 lesions, OPMDs showed clear AFI suppression, with Homogeneous leukoplakia (*n* = 149; GC: 38.62 ± 25.05; BC: 21.60 ± 9.50), non-homogeneous leukoplakia (*n* = 25; GC: 30.42 ± 18.66; BC: 18.25 ± 7.17), oral submucous fibrosis (*n* = 82; GC: 64.13; BC: 28.02), oral lichen planus (*n* = 05; GC: 47.43; BC: 23.06), and oral cancer (*n* = 21; GC: 23.39 ± 15.53; BC: 15.82 ± 7.15; all *p* < 0.001) showed markedly pronounced FVL. Benign lesions showed moderate reductions in green and blue channels (n: 44; GC: 66.99 ± 30.88; BC: 32.01 ± 13.62). Tobacco pouch keratosis demonstrated characteristically elevated red intensity (*n* = 58, Red Channel: 32.30) but substantial reductions in green and blue channels (*n* = 58; GC: 58.41; BC: 24.62), forming a distinct signature (Supplementary Table S3). These quantitative differences were statistically significant across lesion groups (*p* < 0.05), highlight the potential discriminatory capability of AFI in distinguishing normal mucosa from OPMDs and malignancy. The labial mucosa demonstrated significant differences between normal, benign, and OPMD groups; no malignant lesions were recorded in this region (Supplementary Table S4). On the tongue, AFI intensities differed significantly across normal, benign, OPMD, and malignant categories (*p* < 0.05; Supplementary Table S5).

### 3.3. Dimensionality Reduction and Unsupervised Clustering

Principal Component Analysis (PCA) and t-distributed stochastic neighbor embedding (t-SNE) were performed to explore latent structure in the dataset. The analysis included categorical variables (sex, tobacco use, lesion site) and continuous variables (age, mean and standard deviation of RGB intensities for AFI and WLI). Outliers in continuous variables were capped via the K3-sigma method, and categorical variables were transformed into dummy variables.

**Figure 7:**
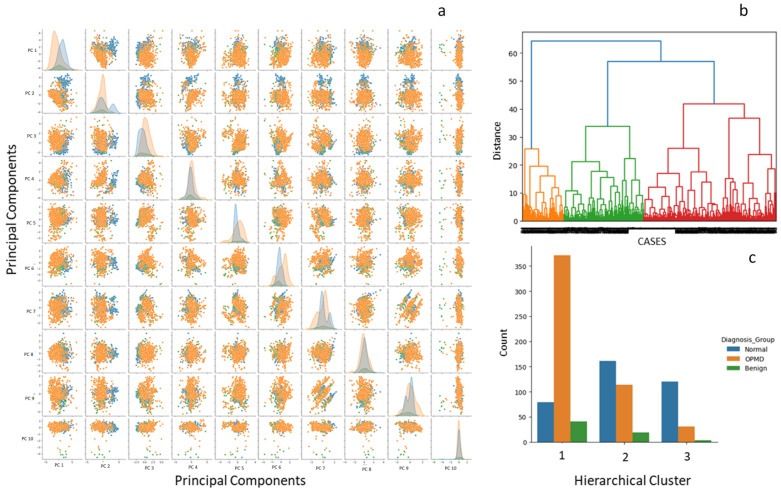
Principal component analysis and hierarchical clustering of intensity features. (**a**) Pairwise distribution of the first ten principal components (PCs). The distribution of PC1 and PC2 shows a clear separation between normal mucosa (orange dots) and OPMD lesions (blue dots). (**b**) Hierarchical clustering based on the PCs identifying three main clusters. The colored branches represent different clusters (orange = cluster 1, green = cluster 2, red = cluster 3). (**c**) Distribution of diagnostic groups across the three clusters and the case distribution of cluster 3 is represented in (**c**) showing OPMD (Oral Potentially Malignant Disorders) predominance.

After standard scaling, the top 10 principal components explained 97% of the total variance (scree plot, Supplementary Figure S4). PCA demonstrated nonlinear separation among diagnostic categories (Figure 7a). Hierarchical clustering using Ward’s linkage and Euclidean distance revealed three major clusters (Figure 7b; Supplementary Figure S5; Supplementary Table S6). Two clusters primarily contained normal and OPMD samples, while the third comprised a heterogeneous mix of both.

**Figure 8:**
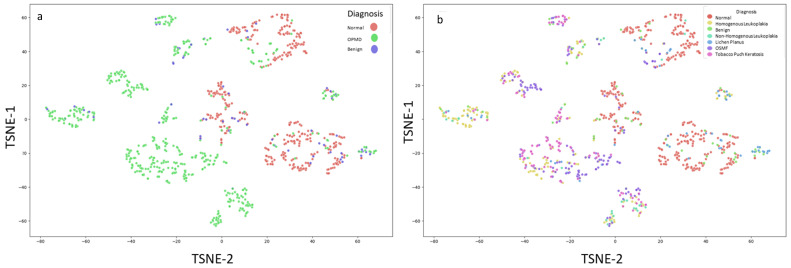
Dimensional reduction using t-Distributed Stochastic Neighbor Embedding (t-SNE). The plot depicts distribution according to diagnosis ((**a**); Normal vs. OPMD). Different OPMD lesion have different distribution (**b**).

For enhanced visualization, t-SNE (perplexity = 10; 1000 iterations) produced two-dimensional embeddings that demonstrated relative clustering of normal and OPMD cases, with additional visual differentiation among OPMD subtypes (Figure 8a,b).

## 4. Discussion

Oral cancer remains one of the leading causes of cancer-related mortality worldwide [13], and early detection through screening is an effective strategy to downstage disease and reduce associated morbidity [15]. Noninvasive imaging methods play a pivotal role in screening and early detection of oral cancer [10]. Several novel optical diagnostic modalities for the oral cavity have become increasingly available to clinicians, offering advantages such as noninvasiveness, patient compliance, real-time data acquisition, repeatability, and high-resolution imaging of both surface and subsurface structures [16]. Among these methods, autofluorescence imaging (AFI) has made a substantial contribution to the noninvasive detection of dysplastic lesions, serving as a valuable adjunct tool for surveillance [13].

Metabolic and structural alterations associated with OPMDs and oral cancer are often accompanied by a FVL or alteration of tissue autofluorescence [17]. There, AFI provides critical clinical insights that complement conventional oral visual examination (COE), supporting lesion detection, risk assessment, and management [18]. However, current AFI approaches rely largely on subjective interpretation, and to date, no study has systematically examined subsite-specific variations in AFI intensity across normal oral mucosa. The present study addressed this gap by quantifying AFI intensity levels across different oral cavity subsites and establishing baseline data for the consistency and reproducibility of the AFI as a point-of-care diagnostic tool over two time intervals. In addition, this study objectively evaluated the utility of the AFI in detecting dysplastic oral lesions, thereby enhancing understanding of both anatomical and optical alterations across subsites.

A dual-mode imaging approach was employed, integrating both WLI and AFI. While WLI enables the clinical assessment of surface alterations, AFI facilitates noninvasive detection of dysplastic lesions on the basis of tissue autofluorescence characteristics. The oral cavity exhibits considerable heterogeneity in mucosal structure, thickness, keratinization, and function [19]. We hypothesize that quantitative assessment of subsite-specific intensity variations is essential for accurate lesion monitoring and for reducing subjectivity in visual interpretation.

Statistically significant differences in mean AFI intensity values were observed between subsites, particularly in the labial mucosa and tongue. The labial mucosa, which is composed of nonkeratinized stratified squamous epithelium, whereas the specialized mucosa of the tongue includes both keratinized and nonkeratinized epithelium, along with lingual papillae and taste buds, these structural differences result in distinct optical properties [14]. Light propagation through these heterogeneous tissue layers is influenced by scattering from randomly distributed optical scatterers, resulting in variability in the AFI signals [20].

Reproducibility of AFI measurements across two time points was evaluated to assess tool consistency and feasibility for surveillance applications.

Overall, intensity values were stable across subsites, except for the labial mucosa, which demonstrated a statistically significant difference at the second visit for WLI. We hypothesize that this discrepancy may be attributed to external factors such as variations in ambient lighting, device settings, or patient positioning. Ensuring controlled imaging conditions with uniform ambient lighting, regular software calibration, slowing performance/speed, and depleting camera protocols may mitigate these effects. Other technical challenges include potential reflections from hard tissues (teeth, restorations, prostheses), lack of standardization in intraoral image acquisition (distance, angulation, retraction), and patient movement during image capture. The unique anatomical features of the labial mucosa, its exposure to the external light, its mobility, curvature, and proximity to ambient light exposure which can affect imaging stability may contribute to increased variability, emphasizing the need for controlled imaging environments.

While AFI demonstrates high sensitivity for detecting dysplasia and cancer, its specificity remains limited because benign inflammatory conditions may also cause reduced autofluorescence. Previous studies have reported 100% sensitivity for AFI when used adjunctively, compared with 17% for COE alone [21]. Prospective studies conducted in both specialist and general dental settings have consistently shown improved sensitivity with AFI relative to COE alone [22,23]. Nonetheless, specificity remains suboptimal, with challenges difficulty distinguishing benign, inflammatory, vascular, or pigmented lesions from dysplastic lesions [17,24,25]. In this context, subsite-specific intensity differences may further influence interpretation, underscoring the need for quantitative AFI analysis.

Interpretation of AFI patterns as FVL or FVR remains subjective, compounded by the lack of standardized techniques and interpretive criteria. Operator training is therefore critical to ensure accurate interpretation and consistency, often cited as a limitation of this modality. Nevertheless, the feasibility of quantitative fluorescence imaging as an objective method for detecting and delineating oral neoplasia has been demonstrated [26]. For instance, one employing quantitative AFI and discriminant analysis reported a specificity of 92.3% and a sensitivity of 97.9% in differentiating cancerous from normal oral mucosa [8]. Another study using multispectral AFI endoscopy combined with machine learning distinguished benign, precancerous, and cancerous lesions with a sensitivity of 85% and specificity of 71% [27].

To our knowledge, no prior study has quantitatively assessed FVL during longitudinal monitoring of OPMDs or oral cancer. In clinical practice, assembling and analyzing serial images of the same lesion is challenging due to differences in the field of view, angle, and perspective across visits. This study addressed these challenges by analyzing the AFI intensity values of normal mucosa and subsequently evaluating benign lesions, OPMDs, and cancerous lesions, thereby establishing a reference framework for tracking lesion progression over time. Objective intensity measurements of AFI can support discrimination between benign, premalignant, and malignant lesions when correlated with anatomical subsites.

Given the dynamic nature of intraoral imaging and inherent variability in acquisition, the imaging protocol was optimized to improve consistency and enable follow-up evaluation. Normalization of the AFI intensity is critical for reducing variability caused by ambient lighting, device-to-device differences, and patient-specific tissue properties. This approach enhances contrast between lesion and normal tissue and facilitates automated lesion classification by thresholding or machine learning approaches [28]. Nonetheless, the use of a prototype device and the imbalance in lesion categories remain potential sources of bias. This imbalance limits the strength of statistical comparisons. Additionally, not all lesions had histopathological confirmation, and clinical diagnosis served as the reference standard in this proof-of-concept phase. Another limitation was the absence of a formal inter-observer and intra-observer repeatability assessment for ROI annotation, which restricted the quantitative reproducibility of the measurements. Image quality was also susceptible to intraoral factors such as saliva, mucosal dryness, and specular reflections. Finally, the uneven distribution of lesion subgroups may affect the generalizability of the findings across the spectrum of OPMDs.

## 5. Conclusions

This phase-1 exploratory study demonstrates the feasibility of quantitative, subsite-specific assessment of normal mucosal autofluorescence variations. The findings provide preliminary observations on lesion-associated intensity changes, providing an initial foundation for future investigation. However, the clinical significance of AFI needs further validation in larger, well-designed cohorts. Additionally, longitudinal studies are needed to better understand the biological properties of different oral lesions, and prospective validation studies with histologically confirmed lesions are warranted.

## Data Availability

The raw data supporting the conclusions of this article will be made available by the authors on request.

## Supplementary Materials

The system does not have any supplementary material files; please add it. The following supporting information can be downloaded at: https://www.mdpi.com/article/10.3390/diagnostics16060857/s1, Table S1: Oral cavity sub-site wise variation in red, green and blue channels mean intensity values for White Light Imaging. Table S2: Oral cavity sub-site wise variation in red, green and blue mean intensity values for Autofluorescence Imaging. Table S3: Lesion-wise depiction of mean intensity values of red, green and blue channels of Autofluorescence Imaging for Buccal mucosa. Table S4: Lesion-wise depiction of mean intensity values of red, green and blue channels of Autofluorescence Imaging for labial mucosa. Table S5: Lesion-wise depiction of mean intensity values of red, green and blue channels of Autofluorescence Imaging for Tongue. Table S6: Silhouette analysis. Figure S1: Paired dot plot showing the mean red, green and blue intensity values for white light imaging and autofluorescence imaging during the first visit and follow-up visit for the buccal mucosa. Figure S2: Paired dot plot showing the mean red, green and blue intensity values for white light imaging and autofluorescence imaging during the first visit and follow-up visit for Tongue. Figure S3: Paired dot plot showing the mean red, green and blue intensity values for white light imaging and autofluorescence imaging during the first visit and follow-up visit for the palate. Figure S4: Principal component analysis showing clusters of normal, benign, and OPMD subtypes. Figure S5: Elbow plot illustrating the change in within-cluster sum of squares (WCSS) as k increases. The rapid drop from k = 2 to k ≈ 4 reflects improved intra-cluster compactness
