# Supplementary material for: Quantitative Autofluorescence Imaging of Oral Mucosa and Lesions: A Proof-of-Concept Study
Source: Diagnostics (Basel). 2026 Mar 13;16(6):857. doi: 10.3390/diagnostics16060857 (PMC13025469; doi:10.3390/diagnostics16060857)
Supplement: Supplementary file 1 [file diagnostics-16-00857-s001.zip › diagnostics-3932114-supplementary.pdf]

Supplementary Tables

RGB channels in White Light Imaging										
**Factor**	**n**	**Red Mean**	**SD**	**Different (P<0.05) from factor nr**	**Green Mean**	**SD**	**Different (P<0.05) from factor nr**	**Blue Mean**	**SD**	**Different (P<0.05) from factor nr**
(1) Buccal Mucosa	120	172.3283	29.3999	(3)(4)(7)(8)	111.5058	13.8797	(3)(5)(7)(8)	71.8272	16.1921	(3)(4)(5)
(2) Floor Of Mouth	60	160.5405	31.6904	(4)	103.0596	17.1545	(4)(5)(6)	69.6096	18.7501	(4)(5)
(3) Gingiva	120	156.8771	30.8589	(1)(4)(5)	92.8106	17.8815	(1)(4)(5)(6)	59.7995	17.9687	(1)(4)(5)(6)
(4) Labial Mucosa	120	186.3703	25.0883	(1)(2)(3)(6)(7)(8)	114.4639	19.3611	(2)(3)(5)(7)(8)	81.2202	19.5143	(1)(2)(3)(5)(7)(8)
(5) Lateral Tongue	120	175.2628	18.8061	(3)(6)(7)(8)	128.8802	17.9597	(1)(2)(3)(4)(6)(7)(8)	97.2267	16.2393	(1)(2)(3)(4)(6)(7)(8)
(6) Palate	60	158.7770	19.7815	(4)(5)	116.5662	13.1149	(2)(3)(5)(7)(8)	73.0475	17.1422	(3)(5)
(7) Retromolar Trigone	30	144.2562	18.1254	(1)(4)(5)	96.9792	16.8527	(1)(4)(5)(6)	63.1505	16.5365	(4)(5)
(8) Ventral Tongue	60	151.8073	23.4645	(1)(4)(5)	100.2144	20.0388	(1)(4)(5)(6)	68.0700	21.0033	(4)(5)

Supplementary table 1 – Oral cavity sub-site wise variation in red, green and blue channels mean intensity values for White Light Imaging

RGB channels in Autofluorescence Imaging										
Factor	n	Red Mean	SD	Different (P<0.05) from factor nr	Green Mean	SD	Different (P<0.05) from factor nr	Blue Mean	SD	Different (P<0.05) from factor nr
(1) Buccal Mucosa	120	14.8577	13.8907	(5)	85.6627	32.2167	(5)	32.3569	8.7287	(5)
(2) Floor Of Mouth	60	12.1350	24.2207	(5)	96.7110	39.6619	(5)(6)(7)	37.1196	11.9007	(5)(6)(7)
(3) Gingiva	120	15.7157	22.8935	(5)	77.6462	36.0367	(8)	31.8487	11.2788	(5)
(4) Labial Mucosa	120	19.8278	23.2137	(5)(8)	91.5149	38.4218	(5)(7)	34.4298	11.6788	(5)
(5) Lateral Tongue	120	37.4152	28.9529	(1)(2)(3)(4)(6)(7)(8)	68.1201	28.2773	(1)(2)(4)(8)	25.2969	7.9335	(1)(2)(3)(4)(8)
(6) Palate	60	7.2472	8.4236	(5)	72.1676	33.4380	(2)(8)	28.8431	9.4190	(2)(8)
(7) Retromolar Trigone	30	17.8845	30.7909	(5)	62.3607	31.3804	(2)(4)(8)	28.2457	13.4084	(2)(8)
(8) Ventral Tongue	60	6.0219	5.9031	(4)(5)	98.8911	42.2220	(3)(5)(6)(7)	37.0814	11.0394	(5)(6)(7)

Supplementary table 2 – Oral cavity sub-site wise variation in red, green and blue mean intensity values for Autofluorescence Imaging

		White Light Imaging		Autofluorescence Imaging	
Lesion	n	Mean Red	Different (P<0.05) from factor nr	Mean Red	Different (P<0.05) from factor nr
(1) Benign	44	159.079	-2	16.9939	
(2) Homogenous_leukoplakia	149	137.272	(1)(5)	10.5821	-8
(3) Malignancy	21	152.368		24.8547	
(4) Non_homogeneous_leukoplakia	25	157.972		10.8653	
(5) Normal	120	172.328	(2)(7)(8)	14.8577	-8
(6) Oral_lichen_planus	40	157.819		9.1404	-8
(7) Oral Submucous Fibrosis	82	141.841	-5	8.2077	-8
(8) Tobacco_pouch_keratosis	58	147.72	-5	32.3004	(2)(5)(6)(7)
Lesion	n	Mean Green	Different (P<0.05) from factor nr	Mean Green	Different (P<0.05) from factor nr
(1) Benign	44	268.23	(5)	66.9954	(2)(3)(4)
(2) Homogenous_leukoplakia	149	262.11	(3) (5)	38.6269	(1)(5)(7)(8)
(3) Malignancy	21	191.00	(2) (5)	23.3946	(1)(5)(7)(8)
(4) Non_homogeneous_leukoplakia	25	211.24	(5)	30.4222	(1)(5)(7)
(5) Normal	120	341.81	(1) (2) (3) (4 (6) (7) (8)	85.6627	(2)(3)(4)(6)(7)(8)
(6) Oral_lichen_planus	40	227.07	(5)	47.4353	-5
(7) Oral Submucous Fibrosis	82	248.51	(5)	64.1358	(2)(3)(4)(5)
(8) Tobacco_pouch_keratosis	58	256.95	(5)	58.4177	(2)(3)(5)
Lesion	n	Mean Blue	Different (P<0.05) from factor nr	Mean Blue	Different (P<0.05) from factor nr
(1) Benign	44	256.34	(5)	32.0122	(2)(3)(4)(6)(8)
(2) Homogenous_leukoplakia	149	26045	(3) (5)	21.6015	(1)(5)(7)
(3) Malignancy	21	287.17	(2) (5)	15.8208	(1)(5)(7)
(4) Non_homogeneous_leukoplakia	25	270.88	(5)	18.251	(1)(5)(7)

(5) Normal	120	300.23	(1) (2) (3) (4 (6) (7) (8)	32.3569	(2)(3)(4)(6)(8)
(6) Oral_lichen_planus	40	257.45	(5)	23.0648	(1)(5)
(7) Oral Submucous Fibrosis	82	258.80	(5)	28.0231	(2)(3)(4)
(8) Tobacco_pouch_keratosis	58	260.24	(5)	24.6213	(1)(5)

Supplementary table 3 – Lesion-wise depiction of mean intensity values of red, green and blue channels of Autofluorescence Imaging for Buccal mucosa

		**White Light Imaging**		**Autofluorescence Imaging**	
Factor	n	Mean Red	Different (P<0.05) from factor nr	Mean Red	Different (P<0.05) from factor nr
(1) BENIGN	13	160.31	(2)(4)(5)(6)	150.46	-5
(2) HOMOGENOUS_LEUKOPLAKIA	15	96.47	(1)(3)	104.5	-6
(3) NORMAL	120	175.3	(2)(4)(5)(6)	127.98	(5)(6)
(4) ORAL_LICHEN_PLANUS	5	81.4	(1)(3)	105.4	
(5) ORAL SUBMUCOUS FIBROSIS	49	67.29	(1)(3)(6)	101.5	(1)(3)(6)
(6) TOBACCO_POUCH_KERATOSIS	56	91.79	(1)(3)(5)	161.24	(2)(3)(5)
Factor	n	Mean Green	Different (P<0.05) from factor nr	Mean Green	Different (P<0.05) from factor nr
(1) BENIGN	13	110.08	-3	108.46	-3

(2) HOMOGENOUS_LEUKOPLAKIA	15	140.93	-6	69.13	(3)(5)
(3) NORMAL	120	157.64	(1)(4)(5)(6)	159.56	(1)(2)(6)
(4) ORAL_LICHEN_PLANUS	5	72.2	-3	107.2	
(5) ORAL SUBMUCOUS FIBROSIS	49	102.55	-3	141.71	(2)(6)
(6) TOBACCO_POUCH_KERATOSIS	56	99.34	(2)(3)	77.45	(3)(5)
Factor	n	Mean Blue	Different (P<0.05) from factor nr	Mean Blue	Different (P<0.05) from factor nr
(1) BENIGN	13	81.81	(2)(3)	133.54	(2)(6)
(2) HOMOGENOUS_LEUKOPLAKIA	15	147.6	-1	72.87	(1)(3)(5)
(3) NORMAL	120	147.95	(1)(5)(6)	157.04	(2)(6)
(4) ORAL_LICHEN_PLANUS	5	98		126	
(5) ORAL SUBMUCOUS FIBROSIS	49	111.35	-3	145.99	(2)(6)
(6) TOBACCO_POUCH_KERATOSIS	56	114.88	-3	70.6	(1)(3)(5)

Supplementary table 4 – Lesion-wise depiction of mean intensity values of red, green and blue channels of Autofluorescence Imaging for labial mucosa

		White Light Imaging		Autofluorescence Imaging	
Factor	n	Mean Red	Different (P<0.05) from factor nr	Mean Red	Different (P<0.05) from factor nr
(1) Benign	6	85.33		59.67	(3)(5)
(2) Homogenous_leukoplakia	7	44	-5	35.14	(3)(4)(5)
(3) Malignancy	9	70.33		107.56	(1)(2)(6)(7)
(4) Non_homogeneous_leukoplakia	7	82.57		84.29	(2)(7)
(5) Normal	120	98.58	(2)(6)(7)	100.58	(1)(2)(6)(7)
(6) Oral_lichen_planus	10	51.7	-5	45.2	(3)(5)
(7) Oral Submucous Fibrosis	15	56.47	-5	36.13	(3)(4)(5)
Factor	n	Mean Green	Different (P<0.05) from factor nr	Mean Green	Different (P<0.05) from factor nr
(1) Benign	6	62.5		22.5	(5)(7)
(2) Homogenous_leukoplakia	7	67.14		66.57	-7
(3) Malignancy	9	67.44		43	(5)(7)
(4) Non_homogeneous_leukoplakia	7	45.86	-5	65.14	(7)
(5) Normal	120	98.38	(4)(6)	92.37	(1)(3)(6)(7)
(6) Oral_lichen_planus	10	49.95	-5	61.8	(5)(7)
(7) Oral Submucous Fibrosis	15	76.47		138.53	(1)(2)(3)(4)(5)(6)

Factor	n	Mean Blue	Different (P<0.05) from factor nr	Mean Blue	Different (P<0.05) from factor nr
(1) Benign	6	61.5		82.83	-7
(2) Homogenous_leukoplakia	7	65.86		93.14	-7
(3) Malignancy	9	72		55.11	-7
(4) Non_homogeneous_leukoplakia	7	29.29	(5)(7)	65.14	-7
(5) Normal	120	99.07	(4)(6)	85.59	-7
(6) Oral_lichen_planus	10	50.1	-5	78	-7
(7) Oral Submucous Fibrosis	15	76.87	-4	138.2	(1)(2)(3)(4)(5)(6)

Supplementary table 5 – Lesion-wise depiction of mean intensity values of red, green and blue channels of Autofluorescence Imaging for Tongue

Clusters	Silhouette score
2	0.1881
3	0.2315
4	0.2480
5	0.2260
6	0.2377

Supplementary Table 6- Silhouette analysis

Supplementary Figures-

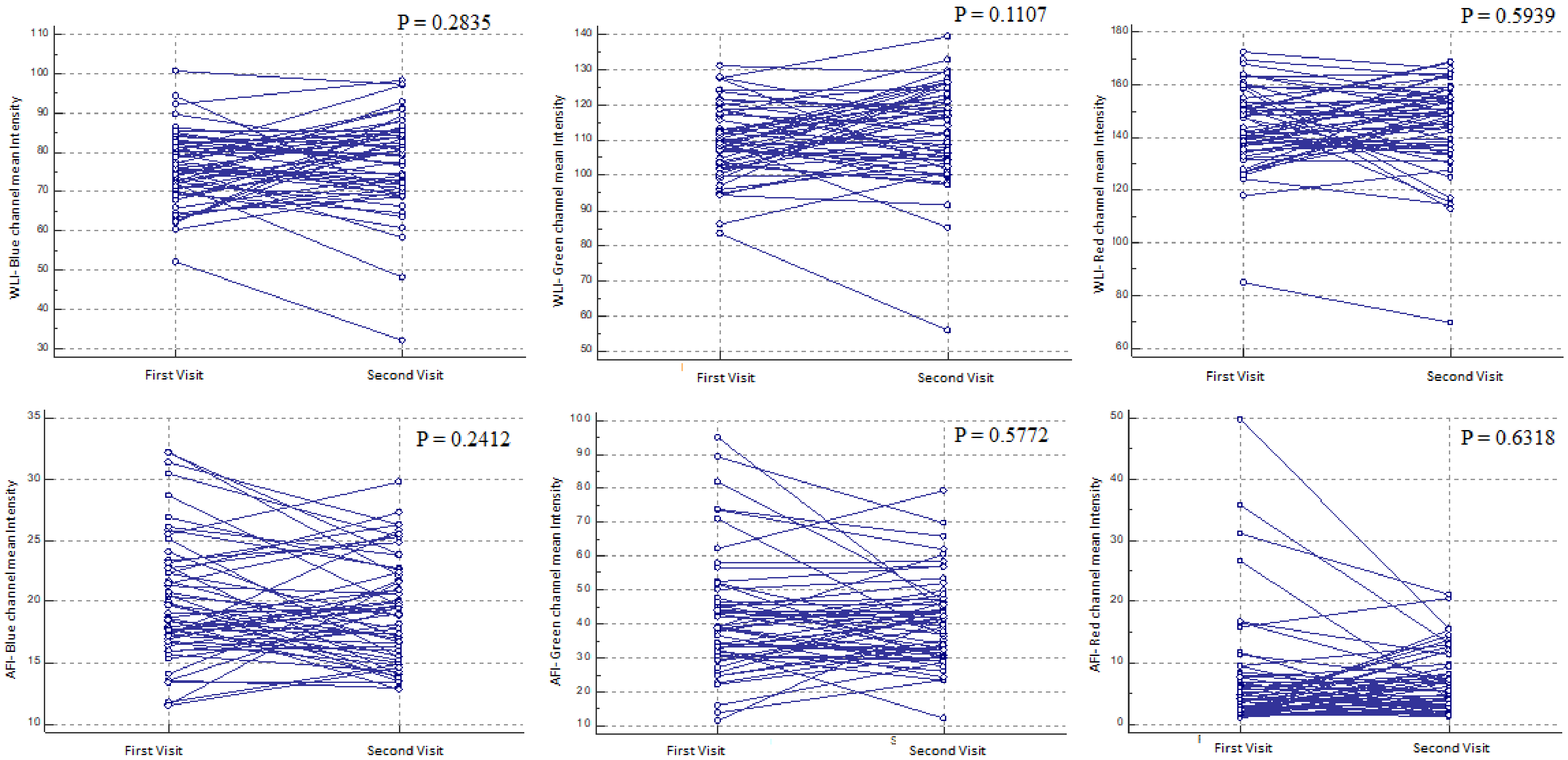

Supplementary Fig. 1- Paired dot plot showing the mean red, green and blue intensity values for white light imaging and autofluorescence imaging during the first visit and follow-up visit for the buccal mucosa.

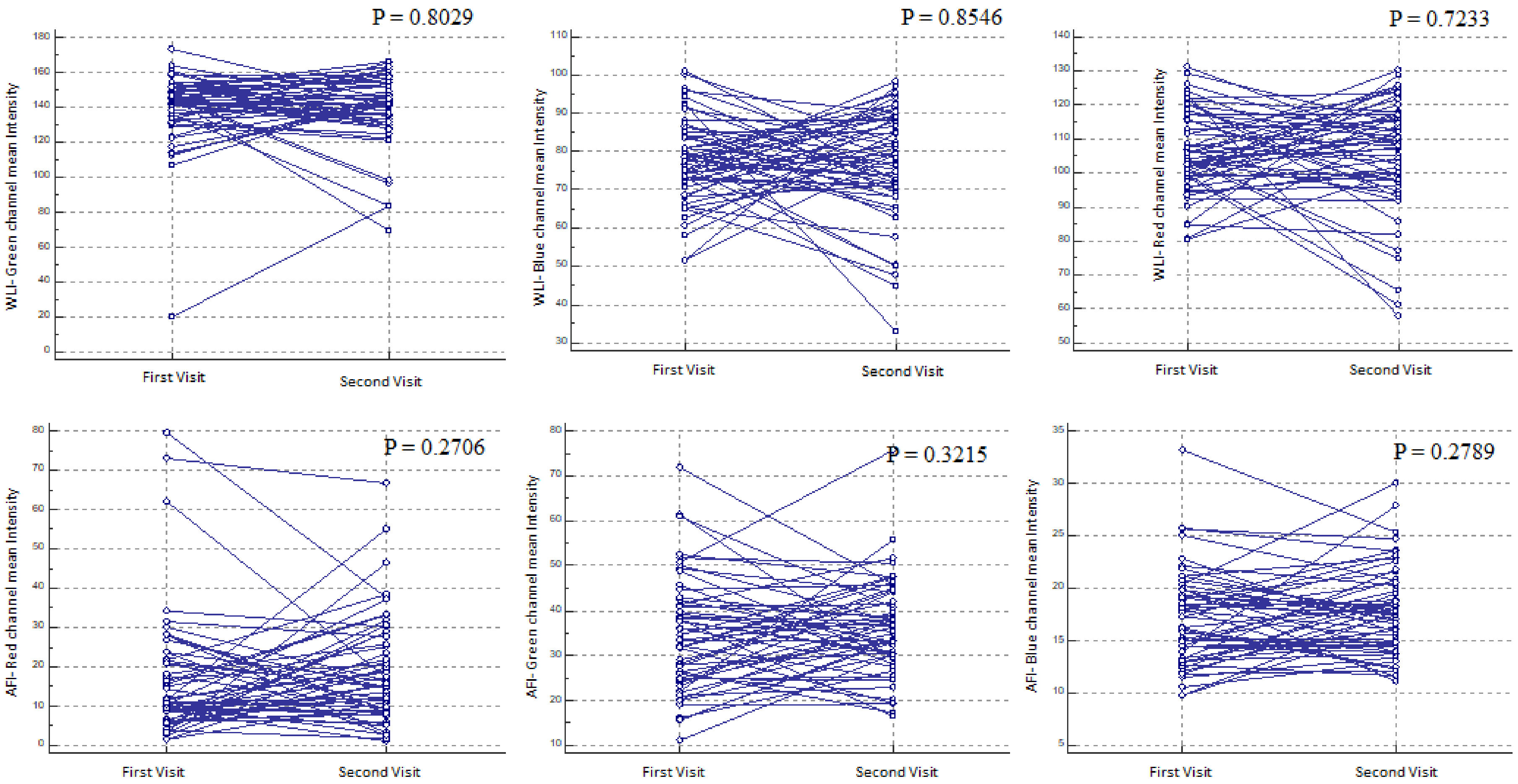

Supplementary Fig 2- Paired dot plot showing the mean red, green and blue intensity values for white light imaging and autofluorescence imaging during the first visit and follow-up visit for Tongue

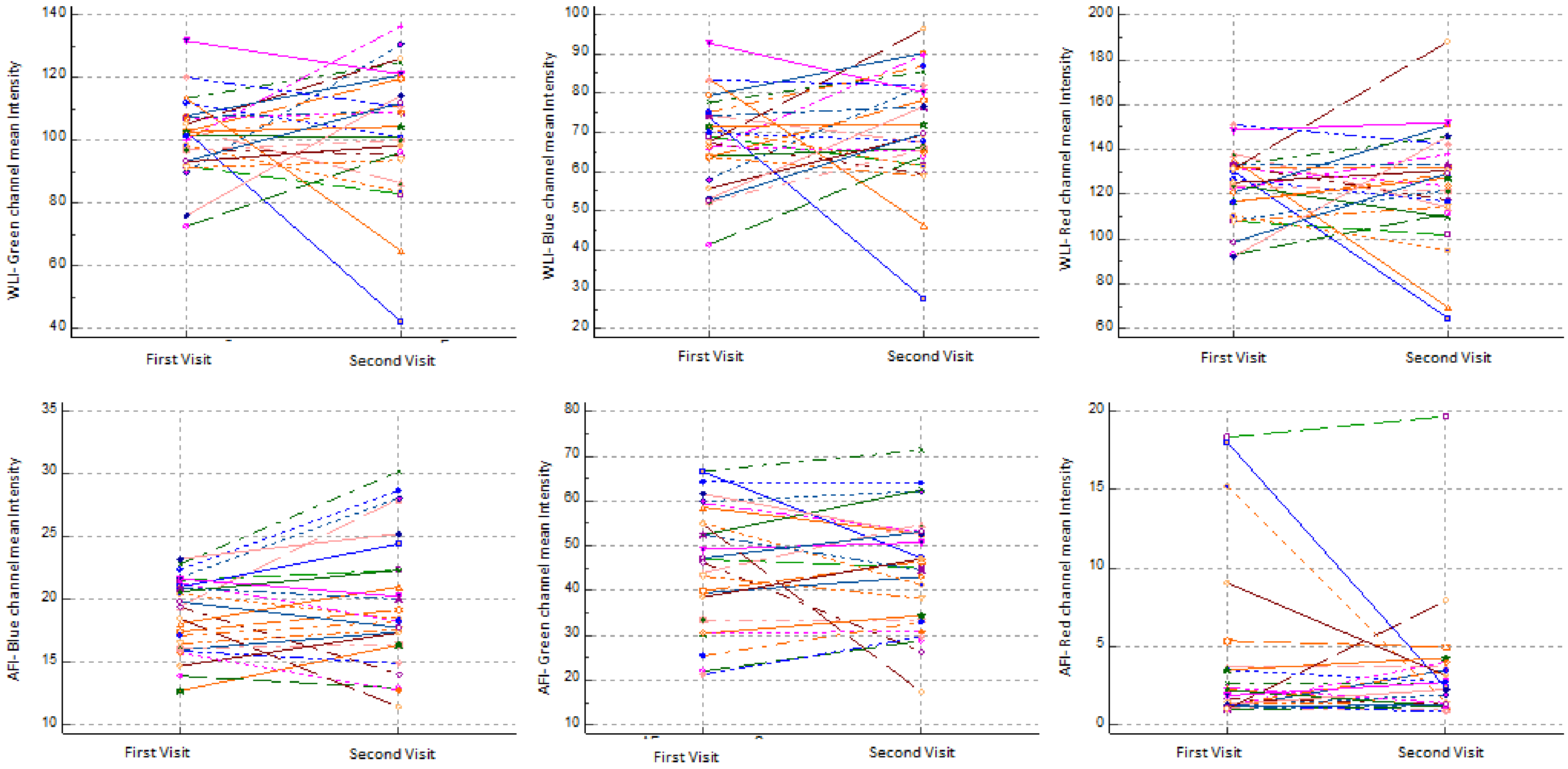

Supplementary Fig 3 - Paired dot plot showing the mean red, green and blue intensity values for white light imaging and autofluorescence imaging during the first visit and follow-up visit for the palate.

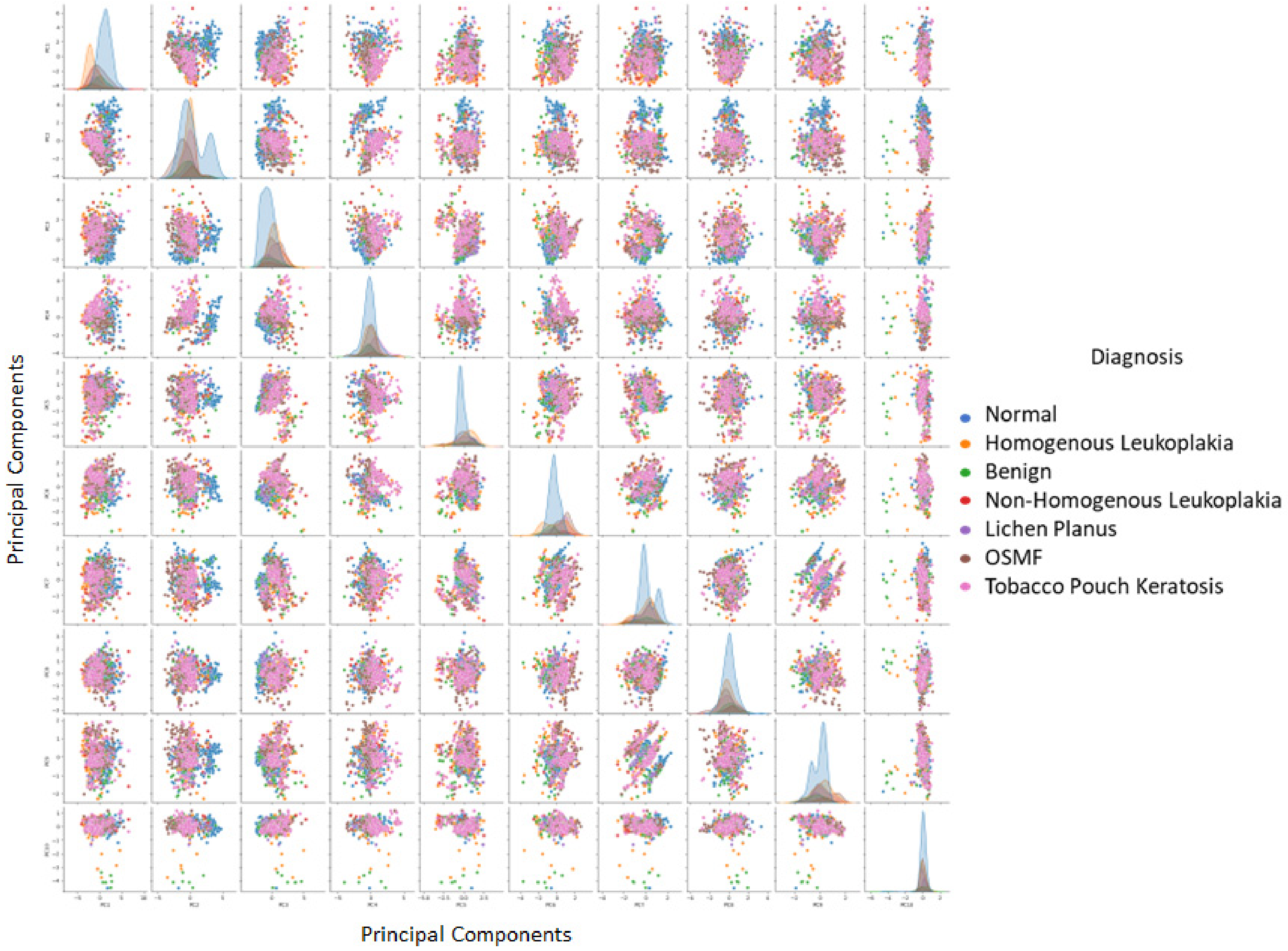

Supplementary Fig. 4- Principal component analysis showing clusters of normal, benign, and OPMD subtypes

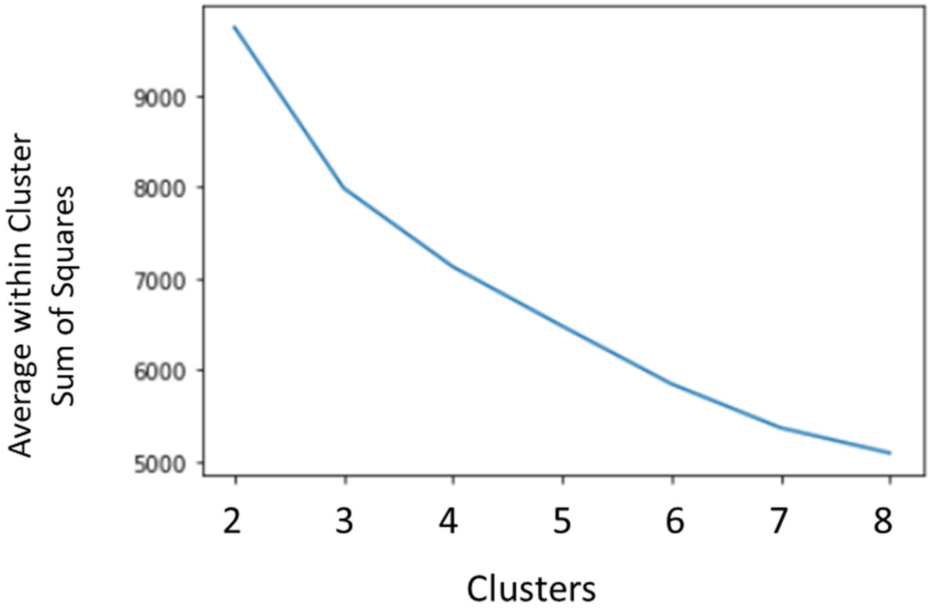

Supplementary Figure 5. Elbow plot illustrating the change in within-cluster sum of squares (WCSS) as k increases. The rapid drop from k = 2 to k ≈ 4 reflects improved intra-cluster compactness.
